# Outcomes between non-IVF and IVF treatment after laparoscopic conservative surgery of advanced endometriosis with Endometriosis Fertility Index score >3

**DOI:** 10.1097/MD.0000000000030602

**Published:** 2022-09-16

**Authors:** Emine Demir, Zeynep Soyman, Sefa Kelekci

**Affiliations:** a İzmir Katip Celebi University, Department of Obstetrics and Gynecology, İzmir, Turkey; b İstanbul Education and Researh Hospital, Department of Obstetrics and Gynecology, İstanbul, Turkey.

**Keywords:** endometriosis, Endometriosis Fertility Index, IVF, laparoscopy

## Abstract

Surgical excision of advanced endometriosis has been demonstrated to improve women’s pain symptoms and quality of life in women in randomized placebo-controlled trials, but there is no strong evidence regarding the live birth rate. The revised American Fertility Society (r-AFS) classification for endometriosis has a limited predictive ability for fertility outcomes after surgery; therefore, EFI scoring has been advised for predicting conception after endometriosis surgery. No randomized controlled trials have investigated fertility outcomes in patients with advanced endometriosis after surgery. This study aimed to determine the outcomes of in vitro fertilization (IVF) or non-IVF treatments after conservative surgery for advanced endometriosis in patients with good prognosis Endometriosis Fertility Index (EFI) scores (>3). This prospective cohort study was conducted between April 2014 and April 2019 at a tertiary research hospital. In total, 113 women with suspected preoperative advanced endometriosis were enrolled in this study. A total of 90 women with advanced endometriosis underwent laparoscopic surgery. Fourteen patients with EFI score of ≤3 and 3 of them who had bilateral tubal occlusion were also excluded. Seventy-three women were included in this study. The remaining 30 women in the non-IVF group and 32 women in the IVF group were analyzed. The main outcome measures were cumulative pregnancy rates and live birth rates in both groups. Women who underwent IVF treatment were older than women (30 ± 3.41) who had non-IVF treatment (26.5 ± 3.07) after laparoscopic surgery (*P* < .001). The remaining baseline characteristics of the patients in both groups were similar. Clinical pregnancy, abortion, and live birth rates were similar in both the groups after 36 months of follow-up. This study demonstrated that cumulative pregnancy and live birth rates were similar to those of non-IVF or IVF treatments after conservative surgery for advanced endometriosis, if patients had good prognosis EFI scores. Furthermore, non-IVF treatments resulted in nearly the same clinical pregnancy results as IVF treatment within 1 year after surgery.

## 1. Introduction

Endometriosis is a common gynecologic condition, affecting 6% to 10% of reproductive-aged women. The most common symptoms are dysmenorrhea, chronic pelvic pain, dyspareunia, and subfertility.^[1]^ Furthermore, endometriosis is also associated with anxiety.^[2]^ Approximately 25% to 50% of infertile women have endometriosis, and 30% to 50% of women with endometriosis are infertile.^[3]^

Today, the most widely used staging system of endometriosis is the revised American Fertility Society (r-AFS) classification. The r-AFS classification is used to predict the recurrence potential of endometriosis after surgery. However, it has limited predictive ability for pregnancy after surgery.^[4–6]^

The Endometriosis Fertility Index (EFI) is used to predict conception after endometriosis surgery.^[7]^ EFI combines conception-related factors such as age, duration of infertility, and gravidity history in addition to providing a detailed score to the adnexa (fallopian tubes, fimbriae of fallopian tubes, ovaries). The EFI contains all of the components of the r-AFS score, but the r-AFS score includes only 20% of the EFI.

Surgical excision of advanced endometriosis has been demonstrated to improve women’s pain symptoms and quality of life in randomized placebo-controlled trials; however, the largest randomized placebo-controlled trial assessed only early stage disease and reported an improvement in live birth rate following surgical excision for fertility outcomes. No randomized controlled trials exist for the fertility outcomes of advanced endometriosis and the data for this group of women is limited.^[8]^ Given the ethical and fiscal limitations of undertaking randomized controlled trials for advanced endometriosis, it is unlikely that these data will ever be available and we must rely on other sources to fully inform women of what their outcomes are likely to be with advanced endometriosis treated surgically.

This study aimed to determine the outcome of IVF (in vitro fertilization) or non-IVF treatments after conservative surgery of advanced endometriosis if patients had good prognosis EFI scores (>3).

## 2. Material and Methods

This prospective cohort study was conducted at a tertiary teaching center between April 2014 and April 2019.

### 2.1. Patient selection

In total, 113 women with suspected endometriosis preoperatively were enrolled in this study. Inclusion criteria were as follows: patients suffering advanced endometriosis and subfertility, age <38 years, first attempt for surgery, preoperative no medical treatment, and no other known cause of subfertility.

The study was carried out in accordance with the Declaration of Helsinki for medical research involving human subjects and approved by the local ethical committee (protocol number 2014-12) and a written informed consent form was obtained from all patients.

### 2.2. Laparoscopic surgery

After detailed preoperative work-up for laparoscopic surgery, complete excision of all endometriotic lesions using bipolar energy with maximum respect for the preservation and/or reconstruction of reproductive anatomy. If endometriomas were present, total cystectomies were performed. The recto-sigmoid colon was mobilized. Bilateral ureterolysis was then carried out. The pouch of Douglas and pararectal spaces were identified and opened. Tubal latency was checked in all patients with methylene blue intraoperatively and all cases had a histological diagnosis of endometriosis.

Exclusion criteria included minimal and mild endometriosis, EFI score ≤3, other causes of subfertility, loss of follow-up, and incomplete recording.

Twenty-three women diagnosed intraoperatively as having mild or moderate endometriosis were excluded. Ninety women with advanced endometriosis have undergone laparoscopic surgery. Fourteen of them whose EFI score was ≤3 and 3 of them who had bilateral tubal occlusion were also excluded. Seventy-three women consisted study group.

All patients were given detailed counseling related to pregnancy potential based on maternal age, treatment modalities, baseline follicle-stimulating hormone levels, and anti-Müllerian hormone measurements blindly to their EFI scores. All selected patients wanted to become pregnant immediately. The women opted independently for IVF or non-IVF management after detailed counseling (self-decision). None of the fertility management decisions were based on the patients’ EFI scores who had an EFI score >3.

**Figure 1.**
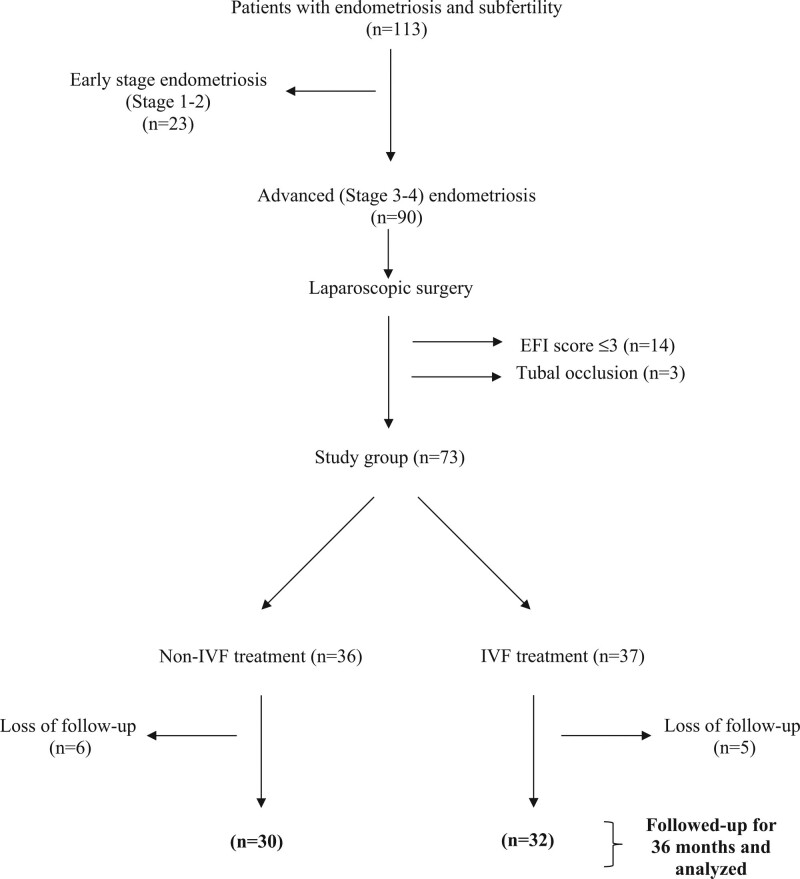
Flowchart of the study.

The non-IVF group consisted of 36 women and the IVF group consisted of 37 women. Eleven women were lost follow-up. The remaining 30 women in the non-IVF group and 32 women in the IVF group were analyzed. The flowchart of patient allocation has been demonstrated in Figure 1.

### 2.3. EFI scoring

**Figure 2.**
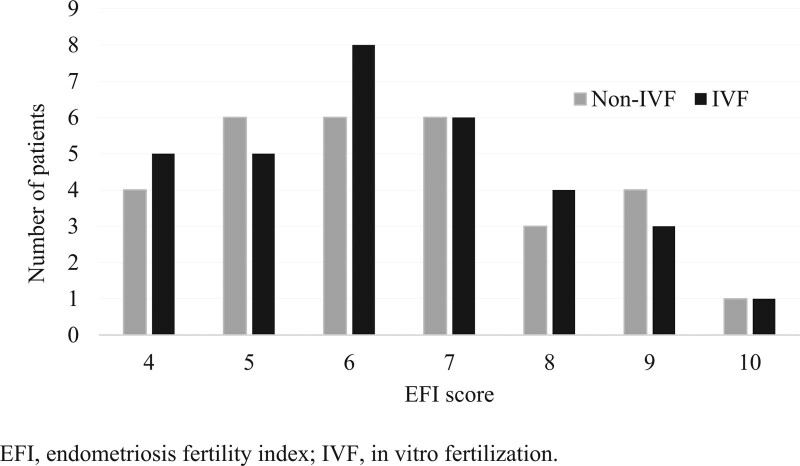
Distribution of patients according to their EFI scores in both groups. EFI = Endometriosis Fertility Index.

The EFI score was calculated according to the EFI scoring system developed by Adamson and Pasta.^[6]^ It includes the following clinical and surgical factors (age, duration of infertility [years], pregnancy history, least-function [LF] score (including fallopian tubes, tubal fimbriae, and ovaries; LF score = the least score of the left side + the least score of the right side; if any ovary was absent, the LF score was obtained by doubling the LF score of the contralateral side), r-AFS score of the lesion, and total r-AFS score. Figure 2 demonstrates the distribution of patients according to their EFI scores in both groups.

### 2.4. Why did we choose the cutoff value as 3 and over for EFI score?

Total EFI scores 0, 1, 2, and 3 were defined as extreme scores in the original paper.^[7]^ Women with very low EFI score (<4) were evaluated in the same group in this study. This group had very poor pregnancy outcome. Estimated cumulative pregnancy rate at 1 year was 9.9% ± 6.7% for overall and 11.1% ± 10.5% for the validation sample in the reference EFI study.^[7]^ These values were not changed after 3 years of follow-up. Because of very low pregnancy rates, non-IVF treatments should not be offered to women with low EFI score.

The main outcome measures were the cumulative pregnancy rates and live birth rates in both groups. Clinical pregnancy (pregnancy diagnosed by ultrasound visualization of 1 or more gestational sacs), abortion rate, and live birth rate were counted as fertility outcomes.

Non-IVF pregnancies were defined as conceptions occurring spontaneously, after ovulation induction with timed intercourse and after controlled ovarian stimulation with intrauterine insemination (IUI). Patients attempted on their own for 12 to 18 months, then had clomiphene citrate 100 mg/d from day 3 through day 7 plus timed intercourse for 3 to 6 cycles; after this treatment, if pregnancy did not occur, some had gonadotropins plus IUI treatment for 3 to 6 cycles.

### 2.5. Statistical analysis

Statistical analysis was performed using SPSS (Statistical Package for the Social Sciences) software (SPSS 20.0 for Mac-OS; SPSS Inc. Chicago, IL). The normally distributed data of quantitative variation and continuous variables are reported as the mean ± standard deviation and were compared using the Student *t* test. Comparison between the 2 different management groups was performed with the chi-square test for categorical variables. Mann–Whitney *U* test was used to compare the groups in the term of the time to clinical pregnancy and variables were given as median (minimum–maximum). *P* < .05 was considered statistically significant.

## 3. Results

Thirty women in non-IVF and 32 in IVF treatment were analyzed. Women who underwent IVF treatment were older than women (30 ± 3.41) who had non-IVF treatment (26.5 ± 3.07) after laparoscopic surgery (*P* < .001). The remaining baseline characteristics of the patients in both groups were similar (Table 1). The remaining baseline characteristics of the patients in both groups were similar. Clinical pregnancy, abortion, and live birth rates were similar for both groups after 36 months of follow-up (Table 2). The cumulative live birth rates in both the groups during 36 months of the follow-up period were demonstrated in Table 3.

**Table 1:** Baseline characteristics of the patients in both groups.

	Non-IVF group (N = 30)	IVF group (N = 32)	*P* values
Age (yr)	26.5 ± 3.07	30 ± 3.41	<.001
Body mass index (kg/m^2^)	22.4 ± 5.85	24.7 ± 7.02	.343
Primer subfertility, n (%)	21 (70%)	24 (75%)	.659
FSH (mIU/mL)	6.3 ± 1.51	6.97 ± 1.84	.119
AMH (ng/mL)	3.16 ± 0.66	2.93 ± 1.36	.467
r-AFS score	59 ± 19.48	60 ± 16.61	.432
EFI score	6 ± 1.72	6 ± 1.66	.832
Number of cycles, n	88	97	–

AMH = anti-Müllerian hormone; EFI = endometriosis fertility index, FSH = follicle-stimulating hormone, IVF = in vitro fertilization, r-AFS = revised American Fertility Society.

*P < .05 was considered statistically significant.

**Table 2:** Fertility outcomes of both groups after laparoscopic surgery for 36-mo follow-up.

	Non-IVF group (N = 30)	IVF group (N = 32)	*P* value
Clinical pregnancy, n (%)	12 (40)	19 (59.38)	.127
Abortion rate, n (%)	2 (6.67)	3 (9.38)	.696
Live birth rate, n (%)	10 (33)	16 (50)	.184
Time to clinical pregnancy after surgery (mo), median (min–max)	6 (3–24)	6 (3–36)	.536

IVF = in vitro fertilization.

* *P* < .05 was considered statistically significant.

**Table 3:** Cumulative live birth rates of both groups during 36-mo follow-up.

Duration of follow-up after conservative laparoscopic surgery	Cumulative pregnancy rate in non-IVF group (N = 30)	Cumulative pregnancy rate in IVF group (N = 32)	*P* value
3 mo	10% (n = 3)	15.63% (n = 5)	.509
6 mo	23.33% (n = 7)	28.13% (n = 9)	.667
12 mo	30% (n = 9)	34.38% (n = 11)	.263
24 mo	33.33% (n = 10)	43.75% (n = 14)	.4
36 mo	33.33% (n = 10)	50% (n = 16)	.184

IVF = in vitro fertilization.

## 4. Discussion

Several studies demonstrated that, in infertile women with endometriosis minimal and mild endometriosis, clinicians should perform operative laparoscopy (excision or ablation of endometriosis lesions) including adhesiolysis, rather than performing diagnostic laparoscopy only, since there is a positive effect in regards to live birth (odds ratio 1.64; 95% confidence interval 1.05–2.57).^[9,10]^ There is no randomized controlled trial or meta-analysis to assess whether surgery is positively effective or not on pregnancy rates in moderate to advanced stage endometriosis. Despite the lack of current data, to do nothing to a patient with severe endometriosis who is already under anesthesia, is ethically unacceptable.^[11]^ A nonrandomized study^[12]^ demonstrated that the cumulative probability of pregnancy in 216 infertile patients with severe disease 2 years after surgery was significantly increased. Moreover, a recent study by Bianchi et al^[13]^ looking at women with deep infiltrative endometriosis found that extensive laparoscopic excision of endometriotic lesions improved pregnancy outcomes significantly (odds ratio 2.45). Thus, we excluded patients with early stage endometriosis and focused on the comparison of pregnancy outcomes between non-IVF and IVF treatment after laparoscopic conservative surgery of advanced endometriosis in our study. In addition, we also excluded patients who had a definitive reason for IVF treatment after laparoscopic surgery of severe endometriosis, for instance, advance age, bilateral tubal occlusion, or diminished ovarian reserve.

To date, the most frequently used staging system for endometriosis is the revised American Fertility Society (r-AFS) score (ASRM, 1997).^[14]^ Unfortunately, this classification system has some serious limitations, including not predicting postoperative pregnancy rates in infertile patients.^[6,15,16]^ For this reason, EFI was developed to estimate the natural conception rate after laparoscopic surgery. EFI has also been validated externally in endometriosis-associated infertile patients in Belgium,^[17]^ China,^[5]^ France,^[18]^ and Italy.^[19]^ None of the above studies discussed the pregnancy rates with non-IVF or IVF treatment after laparoscopic surgery of advanced endometriosis for patients with similar EFI scores.

The choice of treatment (expectant management, non-IVF, or IVF) for infertility after laparoscopic surgery was left to individual patient preference according to their personal decision, except for patients who had absolute indications for IVF treatment after laparoscopic surgery in this study. The EFI scores of patients after laparoscopic surgery for both treatment groups were nearly the same (6 ± 1.72 for the non-IVF group and 6 ± 1.66 for the IVF group). As expected, the overall age was older in the IVF group. The ovarian reserve tests were similar in both groups. The live birth rates were also similar for both groups in our study. In a recent study from China, authors found the probability of spontaneous conception as 46.5% after surgery. The pregnancy rate was 54.25% with IVF treatment after surgery in this study.^[1]^ These rates were similar to our results.

In our study, the cumulative pregnancy rates were nearly the same in both groups in 1 year after surgery (43.75% in the IVF group and 43.44% in the non-IVF group). Previously, Lee et al^[20]^ investigated the influence of laparoscopic surgery on the natural conception rate in infertile women with endometriosis during the first year after the surgery. The natural conception rate was 41.9% during the first year after laparoscopic surgery and most of the patients conceived within 6 months after the surgery in this study. These data have been generally reported by other authors. According to results of the above studies and our study, non-IVF treatments could be recommended to patients with advanced endometriosis within 1 year after conservative surgery if they had good prognostic EFI scores. Within additional 2 years of follow-up, 1 year after surgery, we observed that 4 more pregnancies occurred in the IVF group and only 1 pregnancy occurred in the non-IVF group. Somigliana et al^[21]^ observed that delaying conception after surgery was associated with a lower pregnancy rate. Thus, we concluded that non- IVF treatments with at most 1 year should be advised to the patients with advanced endometriosis after conservative surgery.

The major strength of our study is its prospective design. It is also the first study that compared the pregnancy results of non-IVF and IVF treatments after conservative surgery of advanced stage endometriosis if patients had good prognosis EFI scores. All patients were operated by 1 experienced surgeon (S.K.) in the same clinic and all EFI scoring were performed by the same surgeon. The follow-up after surgery was performed by a clinician who was blinded to EFI scores. Patients opted independently for IVF or non-IVF management after surgery with informed decision-making on their own. We think that this situation also prevented the bias depending on patient selection for infertility management after surgery.

There are some limitations in our study: we excluded and did not discuss the results of patients with poor prognosis EFI scores (<4). However, as discussed above, IVF treatment was directly recommended to these patients due to low pregnancy rates that were shown in literature previously. We did not separate non-IVF treatments as expectant management, ovulation induction, or gonadotropins plus IUI treatment because of insufficient patient numbers. We have not evaluated the influence of body mass index with respect to endometriosis grade since the body mass index influence the pregnancy outcomes in endometriosis patients differently in different stage of the disease.^[22]^ Further studies with large patient cohorts could be recommended in this issue.

## 5. Conclusions

Based on the statistical analysis in our study, first, we demonstrated that the cumulative live birth rates were similar with non-IVF or IVF treatments after conservative surgery of advanced endometriosis, if patients had good prognosis EFI scores (>3). Second, non-IVF treatments resulted in nearly the same clinical pregnancy results as IVF treatment within 1 year after surgery. Good prognosis EFI scores could enable targeted and individualized infertility treatment after surgery. This information could be useful to the physicians counseling infertile patients seeking treatment after conservative surgery for advanced stage endometriosis before expensive treatments such as IVF.

## Acknowledgments

Thanks to all the peer reviewers for their opinions and suggestions.

## Author contributions

Conceptualization: Emine Demir, Zeynep Soyman, Sefa Kelekci

Data curation: Emine Demir, Sefa Kelekci

Formal analysis: Zeynep Soyman, Sefa Kelekci

Investigation: Sefa Kelekci

Methodology: Emine Demir, Sefa Kelekci

Software: Emine Demir, Zeynep Soyman

Supervision: Sefa Kelekci

Validation: Emine Demir, Zeynep Soyman, Sefa Kelekci

Visualization: Emine Demir, Zeynep Soyman, Sefa Kelekci

Writing—original draft: Emine Demir, Zeynep Soyman, Sefa Kelekci

Writing—review & editing: Emine Demir, Zeynep Soyman, Sefa Kelekci
